# Effect of Human Testicular Cells Conditioned Medium on *In Vitro* Maturation and Morphology of Mouse Oocytes

**DOI:** 10.22074/ijfs.2020.6097

**Published:** 2020-10-12

**Authors:** Maryam Adib, Seyed Morteza Seifati, Mahmood Dehghani Ashkezari, Fatemeh Akyash, Arezoo Khoradmehr, Behrouz Aflatoonian

**Affiliations:** 1 Medical Biotechnology Research Center, Ashkezar Branch, Islamic Azad University, Ashkezar, Yazd, Iran; 2 Stem Cell Biology Research Center, Yazd Reproductive Sciences Institute, Shahid Sadoughi University of Medical Sciences, Yazd, Iran; 3 Research and Clinical Center for Infertility, Yazd Reproductive Sciences Institute, Shahid Sadoughi University of Medical Sciences, Yazd, Iran; 4 Department of Reproductive Biology, School of Medicine, Shahid Sadoughi University of Medical Sciences, Yazd, Iran; 5 Genetics and Fertility Unit, Erfan Hospital, Tehran, Iran; 6 The Persian Gulf Marine Biotechnology Research Center, The Persian Gulf Biomedical Sciences Research Institute, Bushehr University of Medical Sciences, Bushehr, Iran; 7 Department of Advanced Medical Sciences and Technologies, School of Paramedicine, Shahid Sadoughi University of Medical Sciences, Yazd, Iran

**Keywords:** Conditioned Medium, *In Vitro* Fertilization, *In Vitro* Maturation, Perivitelline Space, Testicular Sperm Extraction

## Abstract

**Background:**

Testicular cell conditioned medium (TCCM) has been shown to induce female germ cell development *in vitro* from embryonic stem cells (ESCs). Testicular cells (TCs) secrete a variety of growth factors such as growth differentiation factor-9 (GDF-9), bone morphogenetic protein 4 (BMP-4), stem cell factor (SCF), leukemia inhibitory factor (LIF), and other, that could improve oocyte maturation. Here we have investigated the effect of human TCCM (hTCCM) on *in vitro* maturation (IVM) and morphology of mouse oocytes.

**Materials and Methods:**

In this experimental study, 360 germinal vesicle (GV) oocytes were obtained from NMRI mice, aged 4-6 weeks that had received 5 IU pregnant mare's serum gonadotropin (PMSG) 48 hours before. GV oocytes were subjected to IVM. 120 GV oocytes were cultured in each medium; hTCCM as the test group, DMEM + 20%FBS as the control group and Ham’s F10 + HFF medium as the sham group. The rates of the IVM and perivi- telline space (PVS) changes were recorded at 8, 16 and 24 hours after culture. The metaphase II (MII) oocytes were subjected for in vitro fertilization (IVF) and the fertilization rate was evaluated after 1, 2, and 3 days.

**Results:**

There was a significant difference between the maturation rates in hTCCM (31.67% MII) and the control [0% MII, P<0.05, (7.5% MI, 52.5% deg. and 40%GV)] groups but there was not a significant difference between the maturation rates in hTCCM and the sham group (53.33% MII, P>0.05). IVF success rate for MII oocytes obtained from IVM in the hTCCM group was 28.94% (n=11). Our data showed that hTCCM is an effective medium for GV oocyte growth and maturation compared to the control medium.

**Conclusion:**

Our findings show that TCCM supports oocyte IVM in mice and affect oocyte morphology.

## Introduction

The last 40 years has witnessed major improvements in curing infertility using assisted conception procedures such as hormonal induction of ovulation; *in vitro* fertilization (IVF), embryo transfer (ET), intracytoplasmic sperm injection (ICSI), and gamete and embryo vitrification. For many patients, the assisted reproductive technologies (ART) can be helpful to treat infertility. Nevertheless, a failure in germ cell development, which is mainly caused by age, disease or a toxic therapy (e.g. chemotherapy), providing an actual treatment is often difficult for the clinicians (1). *In vitro* maturation (IVM) of germinal vesicle (GV) oocytes is an effective method to supply mature oocytes. This method, as a helpful treatment for infertility, is of higher up importance for ART. IVM is a cost-effective and simple treatment for certain infertile couples with few side effects for gonadotropin stimulation (2).

Successful pregnancy and live birth rates fter intracytoplasmic sperm injection of *in vitro* matured GV oocytes was originally reported in 1996 (3). The main challenge in IVM is the preparation of an adequate medium, which provides the most similar microenvironment to the in vivo condition (4, 5). Recent studies have shown that the conditioned medium (CM) from mesenchymal and embryonic stem cells (MSCs and ESCs, respectively) used for IVM, can significantly improve the oocyte maturation and embryo development rates (6, 7, 8).

The preliminary data has shown the effects of testicular cell conditioned medium (TCCM) from rat testis on *in vivo* PGC differentiation of MSCs (9). Furthermore, it was reported that co-culture of ESCs derived primordial germ cell-like cells (PGCLCs) with testicular somatic cells and sequential exposure to morphogens and sex hormones mimics key marks of meiosis (10). Moreover, microarray analysis showed that sertoli cell conditioned medium (SCCM), which contains effective factors for *in vitro* germ cell differentiation could facilitate germ cell progression in human ESCs (hESCs) (11,12). All of these findings reveal the fact that TCCM contains growth factors secreted from various sources of testicular cells (13, 14) that affect PGC formation and meiotic accomplishment in spermatogenesis.

Interestingly there are other reports on the effects of TCCM on *in vitro* oogenesis using ESCs in mouse, buffalo and human (14-16). In 2006, Lacham-Kaplan and co-workers claimed the formation of artificial ovaries containing oocyte-like structures from mouse embryonic stem cells (mESCs) following culture with TCCM (14). Later, in 2016 the supportive effect of the conditioned medium from testicular cells to provide a better female germ cell developmental niche was shown using buffalo ESCs and the gene expression profile assessment of the differentiated cells (15). In summary as IVM is the final part of the *in vitro* oogenesis, these reports indicate the supportive effect of TCCM on *in vitro* oogenesis using gene expression profile assessments, which means there are supportive elements or growth factors within TCCM which can be used in IVM, too.

These findings indicate that factors secreted by testicular cells such as BMP, SCF, epidermal growth factor (EGF), insulin growth factor (IGF), growth differentiation factor-9 (GDF-9) and many others growth factors and cytokines support female germ cell development in mammals (14-15). According to previous studies, it has been proven these factors are also involved in oocyte growth and maturation (16-20), therefore we hypothesized that CM obtained from TESE-derived cell cultures may improve IVM. The present study is the first report to investigate whether human testicular cell conditioned medium (hTCCM) can improve the IVM in mice based on the reports of *in vitro* oogenesis using TCCM.

## Materials and Methods

### Preparation of human testicular cell conditioned medium

In this experimental study, human TCCM was collected from TESE cell cultures as explained elsewhere (21). TESE samples that contained sperm from individuals with non-obstructive azoospermia disorder were used after fully-informed patient consent. This study has 2 Ethical numbers: 1. IR.SSU.REC.1394.102 is for the testicular cell conditioned medium, and 2. IR.SSU.REC.1397.087 is for the IVM part of the project using testicular conditioned medium). After washing the TESE samples in Dulbecco's Modified Eagle Medium + 20% fetal bovine serum (DMEM + 20% FBS, Invitrogen, UK)) medium, tissue fragments were minced into small pieces mechanically with a 19-gauge needle, following enzymatic digestion and were pelleted by centrifugation for 3 minutes at 200 g. The supernatant was removed and the pellet was seeded in tissue culture flasks containing DMEM + 20% FBS medium. Collection of the CM was performed 4 days after each passage with 80-90% confluency, then filtered through 0.22-mm syringe filter and stored at -20°C (13).

### Animals

The Naval Medical Research Institute (NMRI) mice from Yazd Reproductive Sciences Institute animal house were used for gamete collection for IVM and IVF. Mice were maintained on a 12 hours light/12 hours dark cycle, a temperature range of 22-25°C, 40-60% humidity and free access to food and water. All animals were treated according to the ethical guidelines provided by the Yazd Reproductive Sciences Institute Ethical Committee for animal studies.

### Collection of germinal vesicle oocytes and their *in vitro* maturation

**Fig.1:**
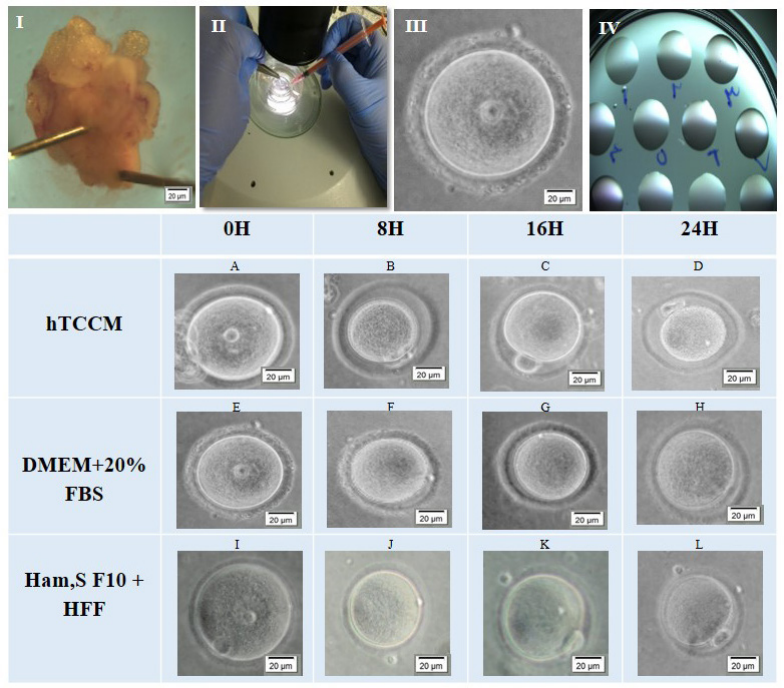
Egg retrieval process in mouse for IVM. **I.** First ovaries were obtained from stimulated mice. **II.** Using the stereo microscope ovary was scratched.** III.** Each individual GV oocyte was recovered and** IV.** Then cultured separately in a small microdrop for IVM. The GV oocyte within each drop was coded for the follow up and data analysis. IVM progress steps at different times in hTCCM and control and sham groups. After 8, 16 and 24 hours in hTCCM and sham condition, some of the GV oocytes **(A, I)** developed further and become mature MII oocytes **(B-D, K-L).** Nevertheless, in DMEM + 20% FBS condition GV oocytes **(E)** could develop to MI oocytes **(F-H)** but none of them could complete the IVM process to form MII oocytes. IVM; *In vitro* maturation, GV; Germinal vesicle, hTCCM; Human testicular cell conditioned medium, MII; Metaphase II, DMEM; Dulbecco's Modified Eagle Medium, FBS; Fetal bovine serum, and H; Hours.

Fifteen 4-6 weeks old female mice received an injection of 5 IU pregnant mare serum gonadotropin (PMSG). At 48 hours post-injection, immature GV oocytes from the ovaries of these mice were extracted. The GV oocyte retrieval was performed by scratching the ovaries with a sterile 28-gauge needles while visualized under a stereomicroscope (Olympus, Japan; Fig. 1I, II).

GV oocytes (Fig . 1III) were individually cultured in microdrops (Fig . 1IV) of hTCCM, DMEM + 20% FBS (control group), or Ham’s F10 + HFF (sham group). In total, 360 GV oocytes were used in this study (120 per group). GV oocytes were incubated at 37°C in a humidified chamber with 5% CO2 for 24 hours. Oocyte maturation, shape and PVS changes were evaluated at 8, 16, 24 hours by a stereomicroscope. Only those oocytes that displayed distinct first polar bodies were classified as metaphase II (MII) oocytes. The MII oocytes were selected for IVF and embryo development.

### *In vitro* fertilization and embryo development

The developmental potential of the oocytes that reached to the MII stage via IVM in hTCCM was evaluated by IVF. Sperms were taken from the caudal epididymis of mature NMRI males and capacitated for 1 hour at 37°C. MII oocytes were incubated with spermatozoa for 4 hours in GIVF medium (Vitrolife, Sweden). Then, the oocytes were washed to remove extra spermatozoa and then cultured in a microdrop of G1-plus medium (Vitrolife, Sweden) at 37°C in a humidified chamber containing 5% CO_2_ for three days. Their developmental stages were determined by morphological evaluations conducted every 24 hours under a stereomicroscope. Fertilization rate was scored as the number and percentage of 2-cell and 4-cell cleavage embryos observed at 24 and 48 hours after insemination.

### Statistical analysis

Maturation rate, shape and PVS changes and developmental competence in mouse oocytes were evaluated for each developmental stage category and compared between the test and control groups. The data was analyzed according to the two-sample test and statistical analysis was performed using the chi-square test with R V.3.1.0 software. P≤0.05 was considered statistically significant.

## Results

### Effects of hTCCM on the maturation of mouse germinal vesicle oocytes

Murine GV oocytes were cultured in hTCCM (test group, Fig . 1A) and DMEM + 20% FBS (control group, Fig . 1E) and Ham’s F10 + HFF medium (sham group, Fig .1I) for further IVM. The IVM of the mouse oocytes was assessed for 24 hours; specifically, at 8 (Fig. 1B, F, J), 16 (Fig. 1C, G, K), and 24 (Fig. 1D, H, L) hours. Resumption of meiosis from GV to the MII stage was considered to be oocyte IVM. Significant differences were seen in IVM rates between hTCCM at different hours; 8.33% (8 hours), 26.67% (16 hours), and 31.67 % (24 hours) compared to the control medium group after 8, 16, and 24 hours (P<0.05). However, there was no significant difference in IVM rates between hTCCM at different hours compared to the sham group after 8, 16, and 24 hours: 6.66%, 25%, 53.33%, respectively (P>0.05). Table 1 shows the number and percentages of degenerated, MI, and MII oocytes in the test group (hTCCM), control group (DMEM + 20% FBS) and sham group at different hours.

In the three groups some of the GV oocytes were developed further to MI after 8 hours. Interestingly, the number of the degenerated oocytes (Fig . 2A) in hTCCM group (n=21) was less than the control group (n=29), but it was more than the sham group (n=9). The number of MI (Fig . 2B) oocytes in hTCCM group (n=65) was significantly higher than the control group (n=11), but it was less than the sham group (n=68). The number of the MII oocytes (Fig . 2C) in hTCCM (n=10) was also significantly higher than the control group (n=0). It was also higher than the sham group but not significantly (n=8, Table 1).

**Fig.2:**
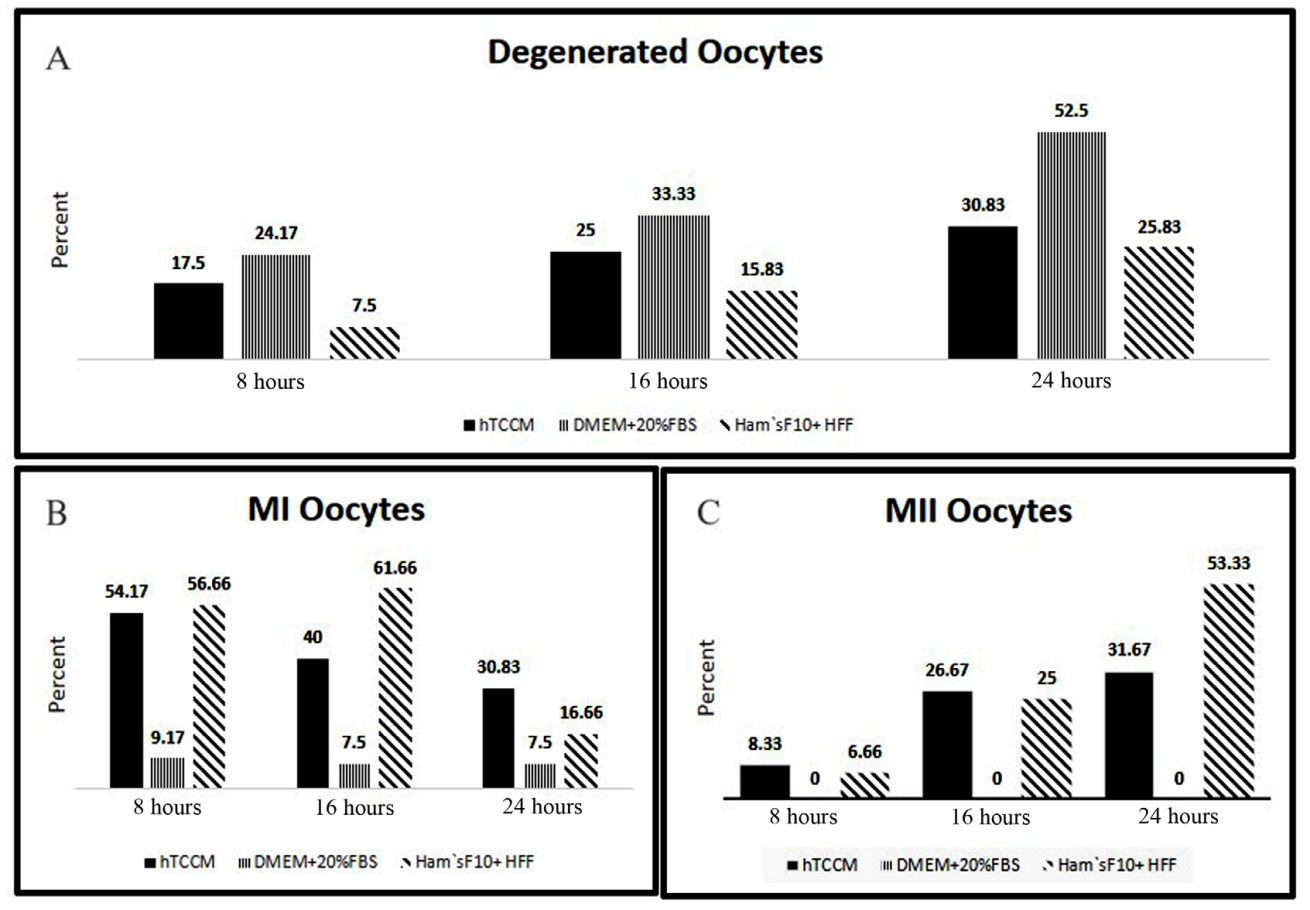
Rate of the degeneration, MI and MII oocytes formation in hTCCM, DMEM + 20% FBS and Ham’s F10 + HFF media after 8, 16 and 24 hours. **A.** Rate of the degeneration increased in three media during 24 hours. The highest rate of the degeneration was in the control medium. **B.** In hTCCM group and Ham’s F10 + HFF medium the number of MI oocytes decreased after 16 and 24 hours due to further maturation to MII oocytes. In the control group MI oocytes decreased after 16 hours due to degeneration. In the control group none of the GV oocytes developed to MII stage.** C.** hTCCM and Ham’s F10 + HFF medium seem to have a supportive progressive effect on the IVM rate to MII stage after 8, 16 and 24 hours. IVM; In vitro maturation, GV; Germinal vesicle, hTCCM; Human testicular cell conditioned medium, MI; Metaphase I, MII; Metaphase II, DMEM; Dulbecco's Modified Eagle Medium, FBS; Fetal bovine serum, HFF; Human follicle fluid, and H; Hours.

After 16 hours, IVM was checked in the three groups and as a result the degenerated oocytes in hTCCM group (n=30) were less than those in the control group (n=40), but it was more than the sham group (n=11, Fig . 2A). Interestingly, the number of MI and MII oocytes in the hTCCM group (n=48 and n=32, respectively) was higher than the control group (n=9 and n=0, respectively), but the number of MI and MII after 16 hours was in the sham group n=74 and n=30, respectively (Fig. 2B, C, Table 1). The degenerated oocytes after 24 hours in hTCCM (n=37) were less than degenerated oocytes in the control group (n=63), but it was higher than sham group (n=31) (Fig . 2A). After 24 hours almost 1/3 of oocytes progressed to MI (n=37) and MII (n=38) in the hTCCM group, whereas in the control group only 9 oocytes and in the sham group 20 oocytes developed further to MI stage. None of the GV oocytes progressed to MII stage in the control group and 64 oocytes reached to MII in the sham group (Fig. 2B, C, Table 1).

To summarize the data, the degeneration rate in the three groups increased after 24 hours. However, the ratio was lower in the hTCCM and sham groups (Fig . 2A). Moreover, all three conditions have favored further development of the GV oocytes to MI stage after 24 hours. This progress was significantly higher in hTCCM group (Fig . 2B). MI formation rates in hTCCM and sham groups have decreasing trends, which might be because of the maturation of the oocytes to MII after 24 hours (Fig . 2C). Also, in the control group MI formation decreased after 24 hours, which was either due to the degeneration or arrest between 16 and 24 hours (Fig . 2B, Table 1). It is noteworthy that the oocyte maturation to MII increased in the test group after 24 hours (Fig . 2C), which indicates the positive effect of time on oocyte IVM in hTCCM (Table 1). Despite development of oocytes to MI stage in the control group, no complete IVM to MII stage happened after 24 hours (Fig . 2C).

**Table 1:** IVM rates at 8, 16, and 24 hours in the three groups


Group	MI n (%) In different time			MII n (%) In different time			Deg. n (%) In different time		
	8 H	16 H	24 H	8 H	16 H	24 H	8 H	16 H	24 H

hTCCM	65 (54.17)	48 (40)	37 (30.83)	10 (8.33)	32 (26.67)	38 (31.67)	21 (17.5)	30 (25)	37 (30.83)
DMEM + 20% FBS	11 (9.17)	9 (7.5)	9 (7.5)	0 (0)	0 (0)	0 (0)	29 (24.17)	40 (33.33)	63 (52.5)
Ham’s F10 + HFF	68 (56.66)	74 (61.66)	20 (16.66)	8 (6.66)	30 (25)	64 (53.33)	9 (7.5)	11 (15.83)	31 (25.83)
^*^ P value		< 0.05			<0.05			0.7	
^**^ P value		< 0.05			0.11			0.1	
^***^ P value		< 0.05			<0.05			0.27	


*; P value between hTCCM and DMEM + 20% FBS, **; P value between hTCCM and Ham’s F10 + HFF medium, ***; P value between Ham’s F10 + HFF medium and DMEM + 20% FBS, H; Hours, n; Number, hTCCM; Human testicular cell conditioned medium, MI; Metaphase I, MII; Metaphase II, Deg.; Degenerated, DMEM; Dulbecco's Modified Eagle Medium, FBS; Fetal bovine serum, HFF; Human follicle fluid, and IVM; *In vitro* maturation.

### Oocyte morphology assessment

Oocyte morphology was evaluated during IVM process under an inverted microscope and was characterized on intra and extra cytoplasmic properties. In this study, we evaluated only some extra cytoplasmic abnormalities: wide PVS and irregular shape. Our results show that there are significant differences in the rates of wide PVS and irregular shapes between the hTCCM and control groups (P<0.05, Fig . 3). Table 2 show number and percentages of oocytes that have wide/normal PVS and irregular/normal shapes following IVM in hTCCM and control groups at different time points.

**Fig.3:**
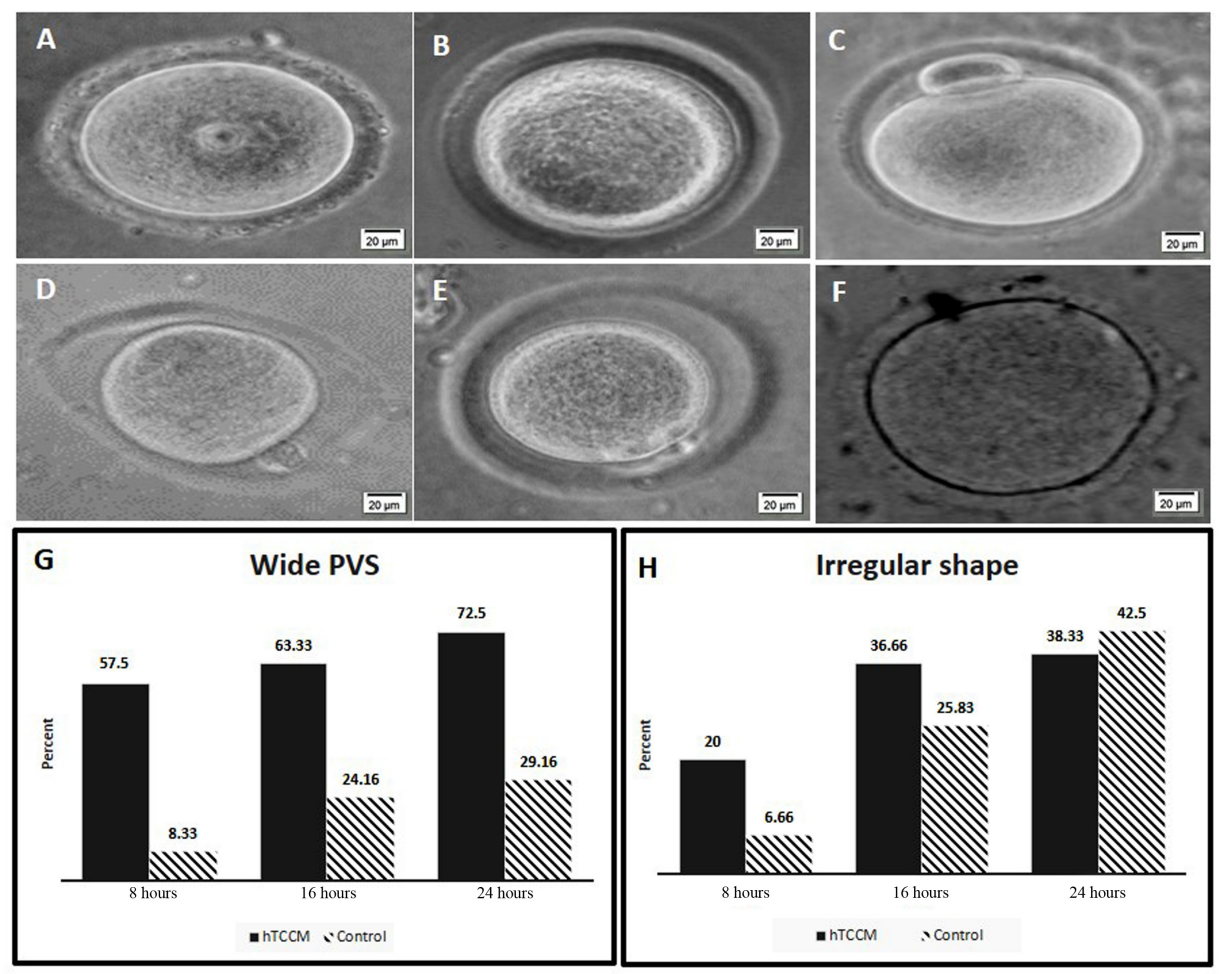
The oocytes shape and PVS change rates in hTCCM and control media during IVM process. **A.** Normal GV, **B.** Normal MI, **C. **Normal MII,** D, E.** Wide PVS, **F. **Irregular shape, and G. Rate of wide PVS oocytes formation during IVM. In hTCCM and control groups the percent of wide PVS oocytes increased after 8, 16 and 24 hours. But this increasing is higher in hTCCM medium and different is significantly. **H.** Rate of irregular oocytes formation during IVM. In hTCCM and control groups the percent of irregular oocytes formation increased after 8, 16, 24 hours and there was significant difference between two groups. PVS; Perivitelline space, IVM; *In vitro* maturation, hTCCM; Human testicular cell conditioned medium, MI; Metaphase I, MII; Metaphase II, and H; Hours.

**Table 2:** Rate of oocytes wide/normal PVS and irregular/normal shape following IVM in hTCCM and control group at different time points


Group	hTCCM			DMEM+20%FBS			P Value
	8 H	16 H	24 H	8 H	16 H	24 H	

Wide PVS n (%)	69 (57.5)	76 (63.33)	87 (72.5)	10 (8.33)	29 (24.16)	35 (29.16)	0.02*
Normal PVS n (%)	51 (42.5)	48 (36.66)	33 (27.5)	110 (91.66)	91 (78.83)	85 (70.83)	0.53
Irregular Shape n (%)	24 (20)	44 36.66)	46 (38.33)	8 (6.66)	31 (25.83)	51 (42.5)	0.02*
Normal Shape n (%)	96 (80)	76 (63.33)	74 (61.66)	112 (93.33)	89 (74.16)	69 (57.5)	0.52


*; Significant level <0.05, H; Hours, n; Number, hTCCM; Human testicular cell conditioned medium, PVS; Perivitelline space, DMEM; Dulbecco's Modified Eagle Medium, FBS; Fetal bovine serum, and IVM; *In vitro* maturation.

### Embryo development following *In vitro* fertilization of *In vitro* maturation oocytes

The developmental competence of oocytes following IVM in the hTCCM group was assessed by IVF and subsequent embryo culture to the 2-cell and 4-cell stages at 24, 48 and 72 hours (Fig . 4A-C). The percentages of 2-cell embryos after 1, 2, and 3 days were 28.94, 34.21, and 28.94, respectively. Moreover, the percentages of 4-cell stage embryos after 1, 2, and 3 days were 10.52, 21.05 and 28.94, respectively.

The developmental rates of embryos to 2-cell stage embryos increased until the second day after IVF, then decreased on the third day. Instead, the number of the 4-cell stage embryos increased up to the third day after IVF (Fig . 4C).

**Fig.4:**
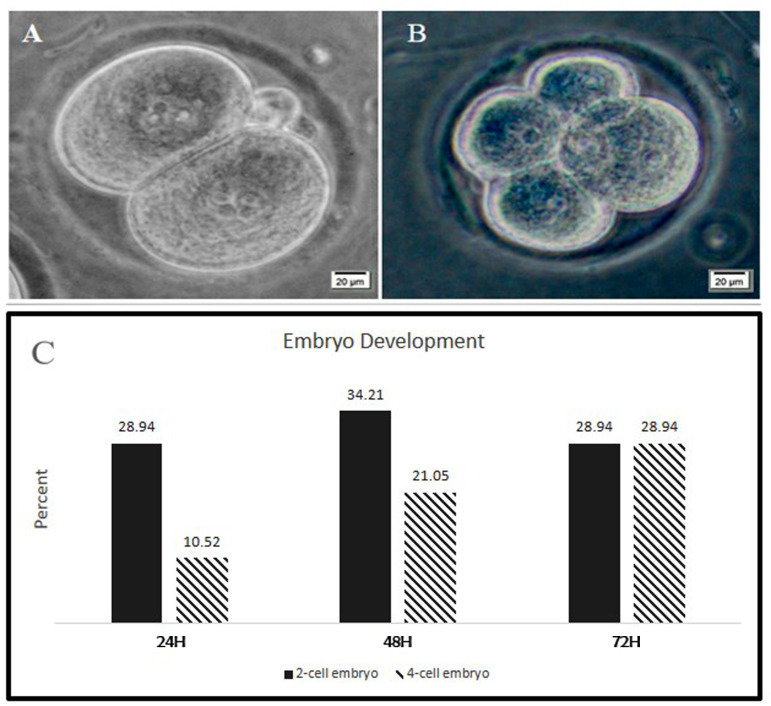
Embryo development following IVF of MII oocytes from the hTCCM group. **A **and **C.** Following IVF from 38 oocytes, after 24 hours, 11 of fertilized oocytes developed to 2 cell embryos and **B and C.** 4 of the oocytes become 4 cell embryos. **A** and **C.** After 48 hours, 13 embryos developed to 2-cell stage embryos and **B and C.** 8 of the MII oocytes formed 4-cells embryos. At 72 hours following IVF, 11 embryos were arrested at 2-cell stage and in total 11 embryos were observed at 4-cell stage. The embryos did not follow further but after three days none of them formed blastocyst. IVF; *In vitro* fertilization, hTCCM; Human testicular cell conditioned medium, MII; Metaphase II, and H; Hours.

## Discussion

This study evaluated the impact of the conditioned media collected from human testicular cell cultures on IVM rates of the GV. The results of this study showed that hTCCM has positive effects on IVM (31.67% MII, n=38) of mouse immature oocytes compared to the DMEM + 20% FBS as the control group [0% MII; (7.5% MI; 52.5% deg. and 40% GV)]. Furthermore, the matured oocytes obtained by IVM in hTCCM were IVF in G-IVF medium, from which 28.94% (n=11) grew and reached to the 4-cell stage embryo in G1 medium.

Human TCs are cultured in DMEM + 20% FBS, thereby, for preparation of hTCCM, the conventional IVM medium (Sage; Cooper Surgical) and recently routine culture medium for IVM (TCM-199; Sigma, USA) were not used in our study. Consequently, DMEM + 20% FBS was used as the control medium and Ham’s F10 + HFF medium was used as the sham medium. Similarly, Ling and colleagues have used DMEM, α-MEM, and HTF as the controls to investigate the effects of mesenchymal stem cell (MSC) conditioned medium on the IVM. Interestingly the rate of IVM in DMEM in their study is higher than HTF (6). Likewise, embryonic stem cell growth medium (ESGM) was used as the control to investigate the effect of the embryonic stem cells conditioned medium (ESCM) on IVM (7). We cannot precisely explain why the results of IVM in our control group (DMEM + 20% FBS) was 0%. Nevertheless, the reason for this difference might be related to the various sources of the serum that were used. In our study, DMEM was supplemented with 20% FBS, whereas Ling et al. used 10% FCS. Moreover, Ling et al. cultured immature oocytes together with granulosa cells, but we did IVM followed by denudation. The other issue might be the effect of group culture for IVM in some studies (7), though single oocyte culture for IVM was done in our study. Further studies are underway using routine standard IVM medium.

About 15% of the oocytes obtained in ovarian stimulation cycles are immature (22). The success rate of pregnancy resulting from embryos of IVM oocytes is very low compared to the embryos that are obtained from immature oocytes resulting from ovulation stimulation (23). Few studies have reported successful fertilization and embryo development from these oocytes that lead to live birth (24). Therefore, many studies have been conducted based on the selection of more suitable factors to modify the culture condition to improve the oocyte IVM efficacy in different species such as porcine (25), bovine (16), and human (26). For instance, it has been shown that by adding some growth factors such as EGF or IGF to IVM medium, the maturity rate and also embryo development rate improve significantly (26, 18, 19). The beneficial effects of EGF on IVM have been demonstrated in different species, including mice (27), humans (28) and deer (29). Similarly, it has been shown that growth and differentiation factor-9 (GDF9), support the folliculogenesis in animal research and also in human organ culture studies (20, 30, 31).

Furthermore, conditioned medium is an important culture supplement device in the IVM process. Several studies have used various types of conditioned medium to improve IVM (31, 32). Similar to our report, cross species studies have indicated the beneficial effect of conditioned medium of one species for IVM of another species (33, 34). It was verified that canine oocytes were able to effectively progress to MII while cultured in bovine cumulus oocyte complex (COC) conditioned medium (34). Similarly, conditioned medium of EC-SOD transgenic mouse embryonic fibroblasts (Tg-CMEF) supports canine oocyte IVM (33). Human bone marrow mesenchymal stem cell (hBM-MSC) conditioned medium was shown to have supportive effect for IVM of mouse oocytes (8).

It has also been shown that IVM using granulosa cell conditioned medium (GCCM) improves the MII oocyte formation rate with a higher expression of genes involved in oocyte maturity (32). Testicular cells are believed to secrete various growth factors that activate signaling pathways finally leading to gametogenesis (13). Moreover using the gene expression profile assessments of the specific markers it was reported that TCCM can support *in vitro* development of ESCs from mouse and buffalo into ovarian structures formation containing oocyte-like cells (14, 15). Thereby, TCCM contain the factors that play a role in oocyte maturation, which can be used to develop a new condition to improve IVM outcomes.

The other issues, which might have an effect on the outcome of IVM are the oocyte retrieval methods and the basal medium used for IVM (35). Here, we have used one method for oocyte retrieval. DMEM+20% FBS was used as basal medium for both control and test groups to keep the condition as consistent as possible during the study.

One of the main determinants of oocyte quality is the morphology of the oocytes, such as: PVS and shape properties (36). Some studies have verified that oocyte morphology has an important role in embryo development (37, 38). Also, it has been informed that great quality embryos are acquired following IVM if normal oocytes are used (38). Perivitelline space anomalies are among the most important abnormalities of the extra cytoplasmic component. It has been suggested that a large PVS may be related to increased oocyte degeneration (38) and lower fertilization rates (39). On the other hand, it was shown that embryo development rate was significantly higher in oocytes that had a PVS abnormality compared to the normal oocytes (40).

This report demonstrates that IVM oocytes cultured in hTCCM may achieve a better meiotic competence and a higher developmental capability than those cultured in DMEM + 20% FBS medium.

## Conclusion

For the first time, our data indicated that hTCCM, which contains putative growth factors, could efficiently improve IVM of mouse GV oocytes. The IVF/IVC of the MII oocytes was assessed for three days until formation of 4-cell stage embryos. Our findings suggest the supportive role of hTCCM in improving IVM conditions as a new insight in infertility treatments.
